# Investigating the potential mechanism of microneedling in alopecia areata mice based on 16S rRNA sequencing and metabolomics

**DOI:** 10.3389/fmicb.2025.1649496

**Published:** 2025-08-20

**Authors:** Yue Zhang, Lu Li, Lei Cao, Rushan Xia

**Affiliations:** ^1^Department of Dermatology, The Affiliated Wuxi No. 2 People’s Hospital of Nanjing Medical University, Wuxi, China; ^2^Department of Dermatology, Wuxi School of Medicine, Jiangnan University, Wuxi, China

**Keywords:** alopecia areata, microneedling, gut microbes, 16S rRNA sequencing, short-chain fatty acids, intestinal microecology

## Abstract

**Background:**

The present study investigates the relationship between alopecia areata (AA) and intestinal microecology, examining the effect of microneedling on the microecology of alopecia areata.

**Methods:**

An animal model of AA was established using imiquimod-induced C3H/HeJ mice. Halometasone was applied topically every 2 days for 2 weeks after a hand-held dermal microneedling treatment. Fecal samples were collected before and after the interventions and underwent 16S rRNA high-throughput sequencing to assess intestinal microecological alterations. Furthermore, changes in short-chain fatty acids (SCFA) associated with AA and microneedling treatment were analyzed.

**Results:**

Microneedling therapy enhanced hair growth in the model group. The model group exhibited a substantial decline in diversity and abundance of gut microbes compared to the control group. After microneedling treatment, the diversity of intestinal microbes was restored, along with a concurrent remodeling of both pathogenic and beneficial bacterial compositions in the model group. In addition, the levels of acetic acid and propanoic acid were elevated in the model group compared to the control group. Following microneedling treatment, a reduction in these levels was observed. In contrast, the model group showed an increase in butanoic acid levels after microneedling treatment; however, this increase did not reach statistical significance.

**Conclusion:**

Microneedling treatment has been shown to improve hair regeneration in AA. Additionally, it positively affects the intestinal microecology related to AA, leading to changes in gut microbes and the production of SCFAs. This provides a foundation for the clinical application of microneedling treatment in AA.

## 1 Introduction

The etiology of alopecia areata (AA) is multifactorial, involving inflammatory pathways, oxidative stress, neuropsychiatric factors, and pathogenic infections. These are often accompanied by comorbidities and micro-ecological imbalances, which may be influenced by a genetic susceptibility in the host (Alessandrini et al., 2021). AA is characterized by the sudden appearance of round or oval patches of hair loss. While most patients with alopecia areata (AA) experience hair regrowth, a substantial number of cases recur and may progress to generalized baldness. This may have a considerable impact on the physical and mental health of the patient.

The microecology of the gut is defined as the ecosystem of microorganisms that inhabit the human gut. These microorganisms interact with the human body, creating a complex ecosystem that significantly contributes to human health. Gut microbes are essential for the development of the intestinal mucosal immune system. The intestinal immune system maintains the stability of gut microbes by forming immune barriers and monitoring the colonization of harmful and conditionally pathogenic bacteria. Numerous extraintestinal diseases are associated with an imbalance in intestinal microecology. The pathogenesis of these diseases involves changes in pathogen-associated molecular patterns (PAMPs), dysfunctional intrinsic immune responses, and dysregulation of Th1/Th2/Th17/Treg cell balance (Cisek et al., 2024; Michaelis et al., 2021; Kwon et al., 2010; Gao et al., 2021). Several studies have shown a correlation between gut microbiota and inflammatory skin conditions, including AA (Mahmud et al., 2022; Sánchez-Pellicer et al., 2022). The diversity and abundance of microorganism flora in fecal samples from patients with AA are diminished relative to healthy individuals (Brzychcy et al., 2022).

Short-chain fatty acids (SCFAs) are metabolites generated through dietary fiber decomposition by the gut bacteria. SCFAs maintain the intestinal barrier, organismal metabolism, immune tolerance, and immune function, influencing innate and acquired immunity (Liu et al., 2023). A fiber-rich diet has been illustrated to enhance the production of SCFAs, subsequently influencing the function of the intestinal epithelium and the immune system (Calabrese et al., 2021). This presents a new approach to the treatment of AA.

Microneedling is a painless and minimally invasive method of drug delivery that involves creating temporary microchannels on the surface of the skin (Waghule et al., 2019). Microneedling stimulates dermal papillae and hair follicle stem cells, enhances blood supply to hair follicles, releases various growth factors, and promotes hair growth (Borde and Åstrand, 2018). Microneedling therapy can offer enhanced efficacy for AA compared to conventional therapy (Almutlq and Bukhari, 2024).

This study aimed to explore the mechanistic link between microneedling treatment of AA and gut microbes and SCFAs. Consequently, we constructed a mouse model of imiquimod-induced AA. Imiquimod stimulates interferon-*α* production by plasma cell-like dendritic cells, which is associated with AA-like alterations in hair follicles of C3H/HeJ mice (Ito et al., 2020). This study aimed to investigate the relationship between alterations in the gut microbiome and AA and the changes in gut flora that occur following microneedling therapy. Additionally, we sought to explore potential mechanistic links between microneedling therapy for AA and gut microbes from a metabolite perspective. Our findings indicate that mice with AA are frequently accompanied by alterations in the composition of the gut flora and its metabolites, namely SCFAs. Furthermore, we observed that microneedling treatment of AA is also associated with changes in the gut flora and SCFAs.

## 2 Materials and methods

### 2.1 Animals studies

The animal experiment plan was approved by the Medical Ethics Committee of Wuxi No. 2 People’s Hospital (2023 Ethical Review No. Y-130, 12 October 2023). 40 SPF-grade, healthy female C3H/HeJ mice were provided by the Human Synergy Biomedical Science and Technology (Wuxi) Co., Ltd. [License No. SYXK (Su) 2022-0048]. The mice were kept in the Animal Experimentation Centre of Wuxi Henghua Science and Technology Park at a temperature of 25°C, with humidity levels of 50–60%, in a relatively quiet environment. They were provided with sterilized feed and clean drinking water. After 1 week of dietary acclimation, the mice were shaved in a portion of the dorsal region of the neck to facilitate the absorption of the topical medication through the skin.

### 2.2 Establishment of the AA mouse model

#### 2.2.1 Grouping

Forty mice were randomly divided into two groups: Ten mice in the control group, which were coated externally with a self-administered blank excipient (a 1:1 mixture of petroleum jelly and lanolin), and 30 mice in the model group, which were coated externally with imiquimod cream.

#### 2.2.2 Modeling method

① Control group: The mice were handled carefully each time. 0.05 g of the blank excipient was uniformly applied to the skin at the collar using a clean medical swab, covering an area of about 1.5 cm × 1.5 cm three times a week (Monday, Wednesday, and Friday) for three consecutive weeks. ② Model group: 0.05 g of imiquimod cream was applied to the skin like the control group. Before each application, a clean medical swab dipped in saline was used to remove any residual drug from the previous application. The application was even, and repeated rubbing was avoided. The hair loss of mice was observed and recorded during the experiment.

Successful modeling was indicated by symptoms such as dry and lusterless fur, loss of appetite, soft stools and diarrhea, depressed spirit, trembling, arched back and curled up, slow response to external stimuli, low activity, and formation of a hair loss spot with an area of >1 cm × 1 cm at the site of drug administration.

#### 2.2.3 Samples collection

**Figure 1:**
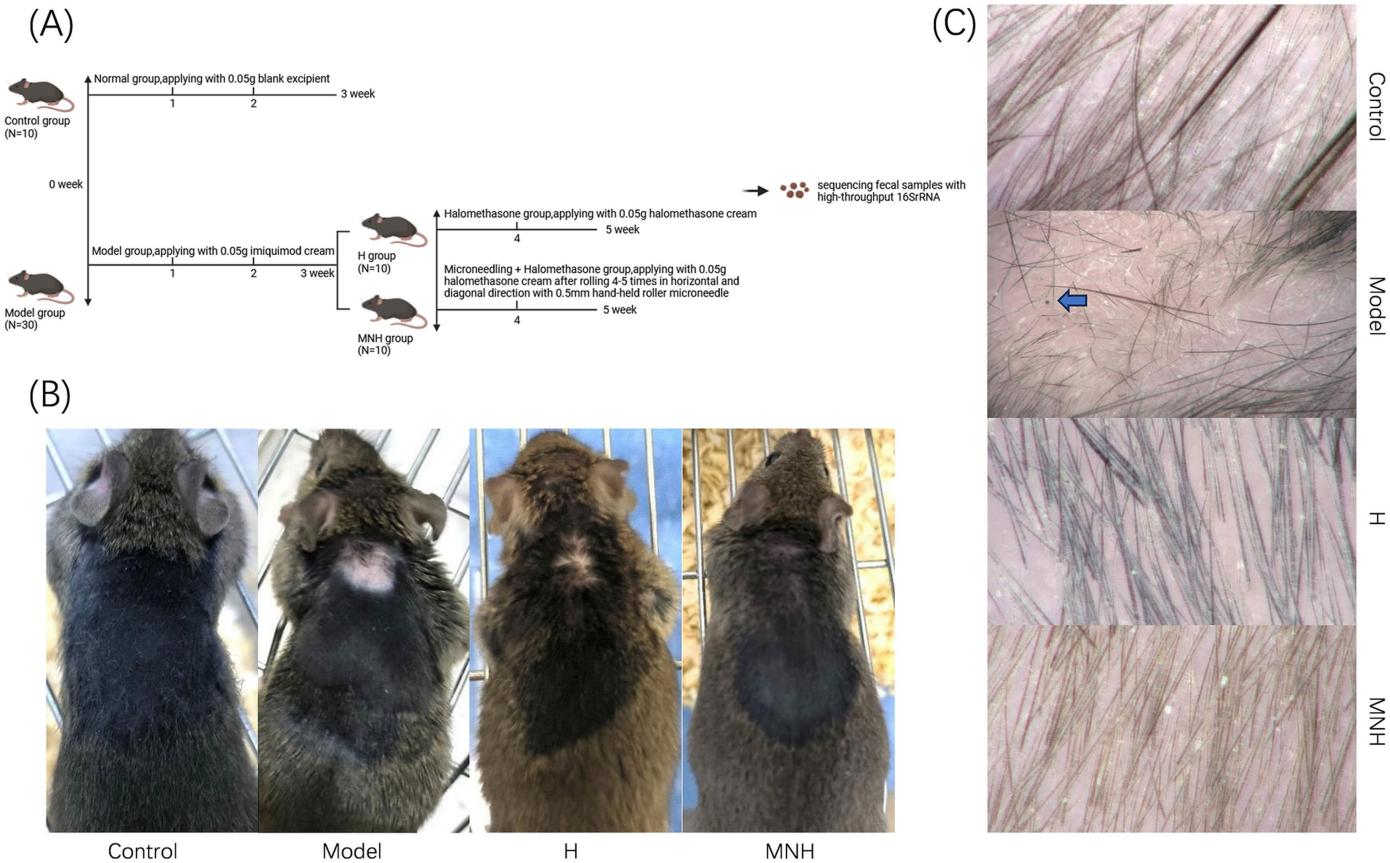
**(A)** Flow chart of experimental mice grouping. **(B)** Skin lesion performance of mice in each group (gross performance). **(C)** Skin lesion performance of mice in each group (hair microscopy performance). The black dot sign and exclamation mark-like hair are visible, as indicated by the arrow in the model group in the figure.

After successful modeling, five mice were randomly selected from each of the control group and the model group; fecal samples were collected, and gross microscopy was performed on the skin lesions of the mice, followed by cervical dislocation and execution of the mice. After successful modeling, ① The remaining 20 mice were randomly divided into 10 mice in the halometasone group (H group) and 10 in the microneedling + halometasone group (MNH group). ② In the H group, 0.05 g of halometasone was applied topically to the skin lesion site of mice once every 2 days for 2 weeks. ③ In the MNH group, after local disinfection of the skin lesion site in mice with alcohol cotton pads, a 0.5 mm hand-held roller microneedle was used to roll and prick the skin lesion along the horizontal and diagonal directions 4–5 times each, followed by topical application of 0.05 g of halometasone to the skin lesion site once every 2 days for two consecutive weeks. ④ Fecal samples were collected from mice in the H group and MNH group after 2 weeks of treatment, respectively. Gross microscopy was performed on the treated areas of the skin lesions of the mice, which were euthanized by cervical dislocation (Figure 1A).

### 2.3 Gross observation and trichoscopic examination of AA mice

The mice were observed and photographed weekly throughout the experiment to document any instances of hair loss. After the modeling and treatment were successfully completed, the mice were euthanized by cervical dislocation, and the skin lesions were examined using trichoscopy.

### 2.4 Fecal samples collection

After modeling and treatment, the mice were transferred to autoclaved cages (no bedding was required) and allowed to defecate naturally. The feces discharged by the mice were collected with sterile tweezers and placed in 1.5 mL sterile EP tubes. The caps of the tubes were carefully closed, and the toothpicks or tweezers were replaced for the next sampling until all the mice’s feces had been collected. A total of four tubes of fecal samples were collected from each group. The fecal samples were immediately transferred to sterile EP tubes and stored at −80°C.

### 2.5 16SrRNA sequencing analysis of the gut microbes

Bacterial DNA was extracted from fecal samples using a DNA extraction kit, and DNA purity and concentration were measured using a NanoDrop 2000 spectrophotometer. The primer pairs (338F: 5′ ACTCCTACGGGGAGGCAGCAG 3′, 806R: 5′ GGACTACHVGGGGTWTCTAAT 3′) were used for the PCR amplification of the highly variable V3–V4 region of the bacterial 16SrRNA gene. The QuantiFluor^™^-ST Blue Fluorescence Quantification System (Promega) quantified the PCR products. They were mixed in proportion according to the sequencing volume required for each sample. The fecal samples of mice were then double-end sequenced by Illumina sequencing.

### 2.6 Measurement of targeted SCFAs

A total of eight SCFAs, including acetic acid, propanoic acid, isobutyric acid, butanoic acid, isovaleric acid, valeric acid, hexanoic acid, and isohexanoic acid were analyzed. The analytical instrument for this experiment was 8890B-5977B from Agilent Technologies Inc., CA (USA). GC/MSD gas chromatograph. The detailed experimental procedures are outlined in the study by Wang et al. (2024).

### 2.7 Statistical analysis

Data results were analyzed using IBM SPSS Statistics 27.0.1 statistical software. A t-test was used to compare the two groups, and a one-way ANOVA was used to compare the multiple groups. The results were presented as mean ± standard deviation (x ± s), with *p* < 0.05 indicating a statistically significant difference between groups.

## 3 Results

### 3.1 Effect of microneedling therapy on skin lesions in AA mice

The impact of microneedling therapy on hair growth in mice with imiquimod-induced AA was examined at both the gross and trichoscopic levels. Hair recovery was significantly more noticeable after receiving microneedling treatment (Figure 1B). Microscopic examination of the mice in the model group revealed lightly reddened skin, accompanied by black dot signs and exclamation mark-like hairs. Following treatment, the skin color was observed to have largely returned to its original state, and normal anagen hairs were noted (Figure 1C). These findings indicated that microneedling treatment may be an effective approach for addressing hair loss associated with AA.

### 3.2 Evaluation of the quality of sequencing data of gut microbes

**Figure 2:**
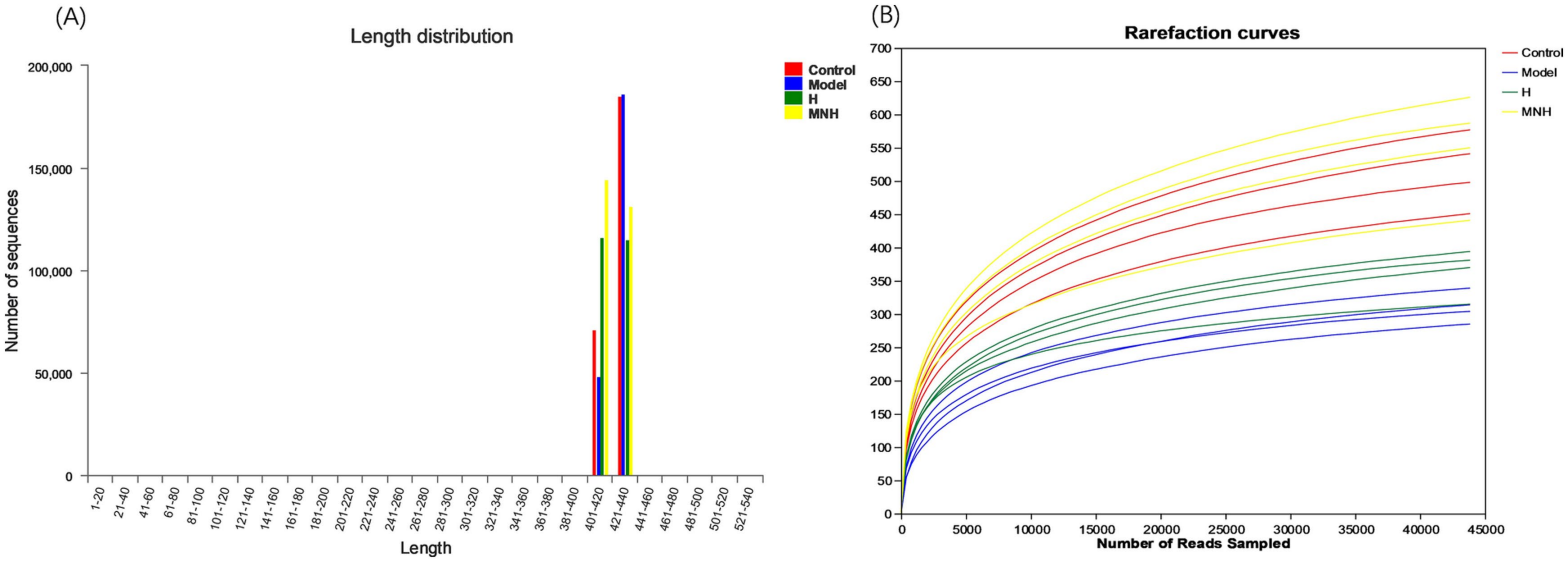
Quality evaluation of mice fecal gut flora sequencing data. **(A)** Sequence length distribution plot. **(B)** Rarefaction curves.

The sequence length data obtained from the sequencing were predominantly 400–440 bp per sample (Figure 2A). As the sample size increased, the number of OTUs climbed more slowly, and the curve gradually leveled off, indicating that the sequencing volume was asymptotically reasonable and that adding more sample sizes would only produce a small number of new species (Figure 2B). This indicated that the number of samples employed in this study was adequate for the investigation.

### 3.3 Effect of microneedling therapy on intestinal OTUs, abundance grading and alpha diversity in AA mice

**Figure 3:**
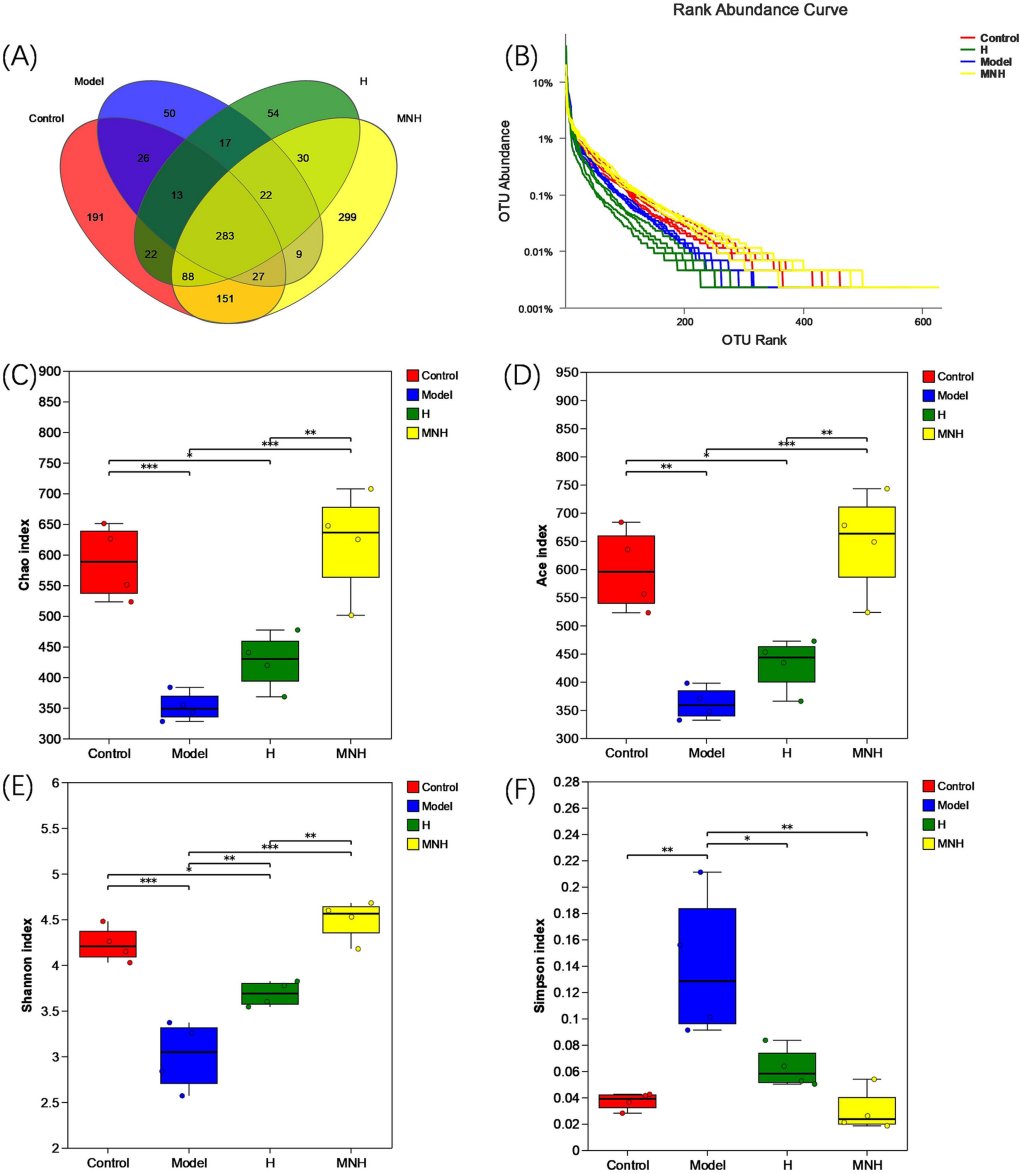
OUT and alpha diversity of mice in each group. **(A)** Number of intestinal OUT of mice. **(B)** Abundance rank curve. **(C)** Chao index. **(D)** Ace index. **(E)** Shannon index. **(F)** Simpson index.

The number of species in the control, model, H, and MNH group was 801, 447, 529, and 909, respectively. Two hundred and eighty-three species were shared at the junction of the four groups (Figure 3A). The decrease in the number of species in the model group compared to the control group indicated that AA could lead to a reduction in the number of gut microbes in mice. The MNH group appeared to have a greater diversity of species compared to the model group, and the gut microbes exhibited a higher species count in the MNH group than those found in the H group. This suggested that microneedling treatment could ameliorate the decrease in the gut microbes caused by AA, and the number of gut microbes was increased compared to the treatment of halometasone alone. Based on the OTU abundance values, the rank abundance distribution curves showed that the MNH group contained the most OTUs (Figure 3B), consistent with the previous results of the Wayne plots. Alpha diversity is a measure that can reflect the diversity, abundance, and homogeneity of the species in the gut microbes (Figures 3C–F). The Chao index and the Ace index are commonly used to reflect the species abundance, and the higher the value, the greater the number of OTUs contained in the colony. The greater the number of OTUs, the higher the richness of the community. As two other indicators to assess diversity, the Shannon index and Simpson index, the higher the value of the Shannon index and the lower the Simpson, the higher the diversity of the community. Compared with the control group, the model group showed a significant decrease in Chao, Ace, and Shannon and a significant increase in Simpson, indicating that AA reduced the diversity and abundance of the gut microbes. The MNH group showed a significant decrease in the Chao index (*p* < 0.001), Ace index (*p* < 0.001), and Shannon index (*p* < 0.001); these were significantly higher than those of the model group. The Simpson index (*p* < 0.01) was significantly lower than that of the model group, and Chao (*p* < 0.01), Ace (*p* < 0.01), and Shannon (*p* < 0.01) were significantly higher in the MNH group than those of the H group, which indicated that microneedling treatment was more able to improve the gut microbial diversity reduction of AA mice than that of halometasone alone. Although the Simpson index indicated that the MNH group also improved the reduction of gut microbes caused by the model group compared to the H group, this result was not statistically significant (*p* > 0.05).

### 3.4 Effect of microneedling therapy on the beta diversity of gut microbes in AA mice

**Figure 4:**
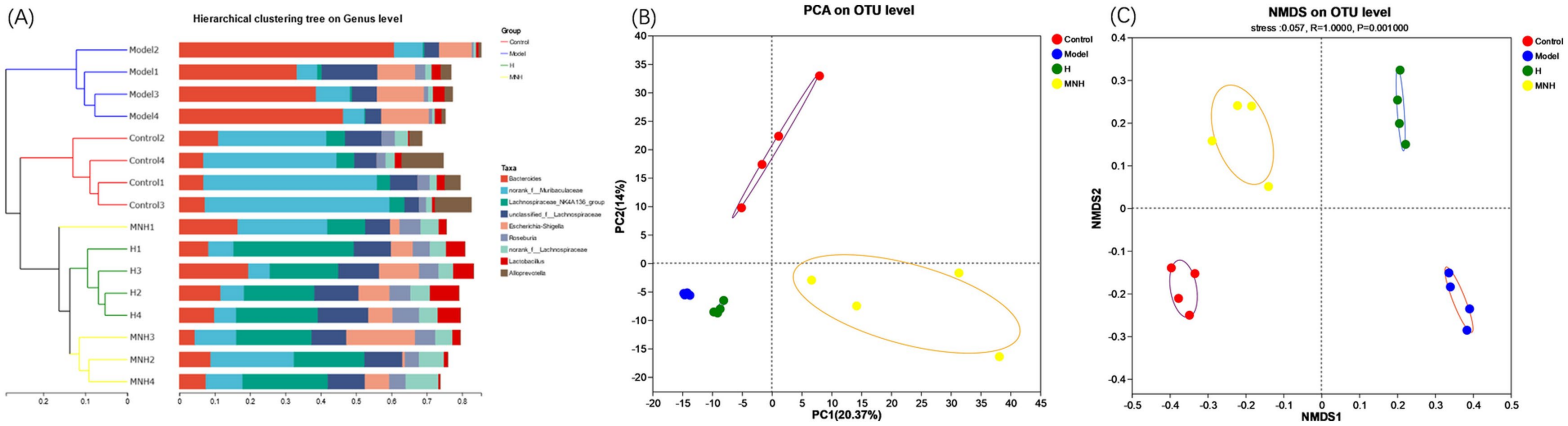
Beta diversity of mice in each group. **(A)** Sample-level clustering tree. **(B)** PCA. **(C)** NMDS.

Beta diversity is frequently employed to reflect the diversity of species within a given group, that is, the degree of variation observed in the sample. Together with alpha diversity, it constitutes the overall diversity of the species. A sample-level clustering tree presents the degree of similarity or difference in communities’ composition in a fecal enteric flora sample. The control and model group were in disparate branches, indicating that the species composition of the two groups exhibited low similarity. Some samples in the control, H, and MNH group were categorized in the same branch. They were situated closer, indicating that the species composition of the three groups was more similar (Figure 4A). The PCA and the NMDS can reflect the differences and distances between the samples. The results demonstrated a notable disparity in the distance between the gut microbe structures of mice in the model and control group (Figures 4B,C), indicating that AA has the potential to alter the gut microbe structure of mice. Following microneedling treatment, the similarity of the community structure was more remarkable in the MNH, H, and control group. The results of beta diversity analysis demonstrated that the similarity of the species composition before and after modeling was minimal. In contrast, the species composition tended to align with the pre-modeling group following treatment. These indicated that microneedling treatment can enhance intestinal bacterial structure and restore it to a normal state.

### 3.5 Effect of microneedling therapy on the relative abundance of gut microbes in AA mice

**Figure 5:**
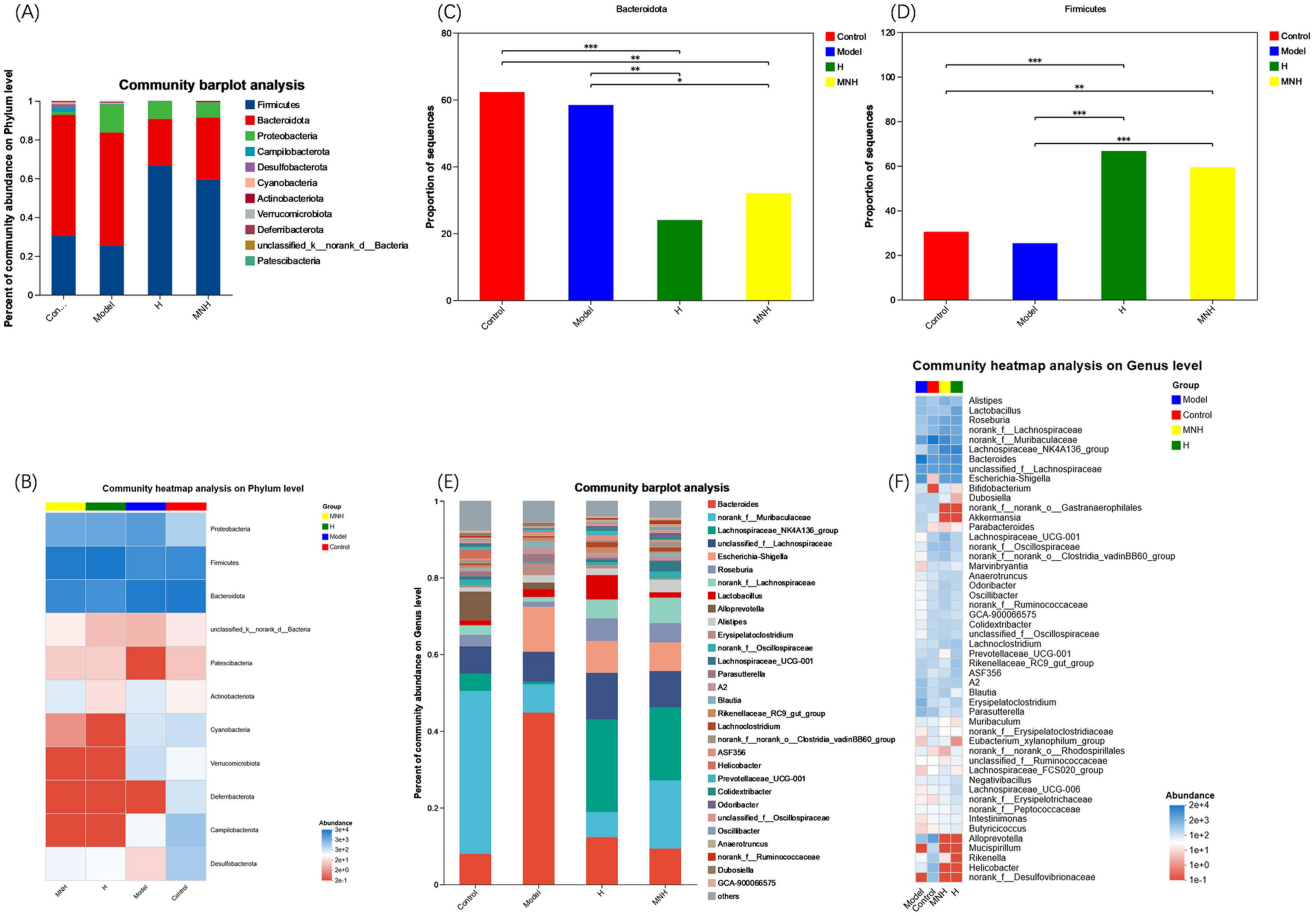
Comparison of mean abundance of species in the gut flora across groups. **(A)** Histogram of the mean abundance of species at the phylum level. **(B)** Heatmap of the mean abundance of species at the phylum level. **(C)** Firmicutes. **(D)** Bacteroidota. **(E)** Histogram of the mean abundance of species at the genus level. **(F)** Heatmap of the mean abundance of species at the genus level.

At the phylum level, Firmicutes and Bacteroidota had the highest fecal gut microbial abundance in all groups (Figures 5A,B). The relative mean abundance of Firmicutes was lower in the model group (0.252987) than in the control group (0.304735), and higher in the H and MNH group (0.666726, 0.593964) than in the model group (0.252987). The relative mean abundance of Bacteroidota was higher in the model group (0.583816) than in the control group (0.622737), and lower in the H and MNH groups (0.239869/0.320060) than in the model group (0.583816). The F/B value was lower in the model group than in the control group and increased after microneedling treatment (Figures 5C,D). This suggested that microneedling therapy inhibits harmful bacteria and has a relatively upregulating effect on beneficial bacteria.

At the genus level, the top 10 genera with high species abundance in each group were Bacteroides, norank_f__Muribaculaceae, Lachnospiraceae_NK4A136_group, unclassified_f__Lachnospiraceae, Escherichia-Shigella, Roseburia, norank_f__Lachnospiraceae, Lactobacillus, Alloprevotella, and Alistipes (Figures 5E,F). The comparison of the average relative abundance of genera in each group is listed as follows: ① Bacteroides: Model group > H group > MNH group > Control group; ② norank_f__Muribaculaceae: Control group > MNH group > Model group > H group; ③ Lachnospiraceae_NK4A136_group: H group > MNH group > Control group > Model group; ④ unclassified_f__Lachnospiraceae: H group > MNH group > Model group > Control group; ⑤ Escherichia-Shigella: Model group > H group > MNH group > Control group.

**Figure 6:**
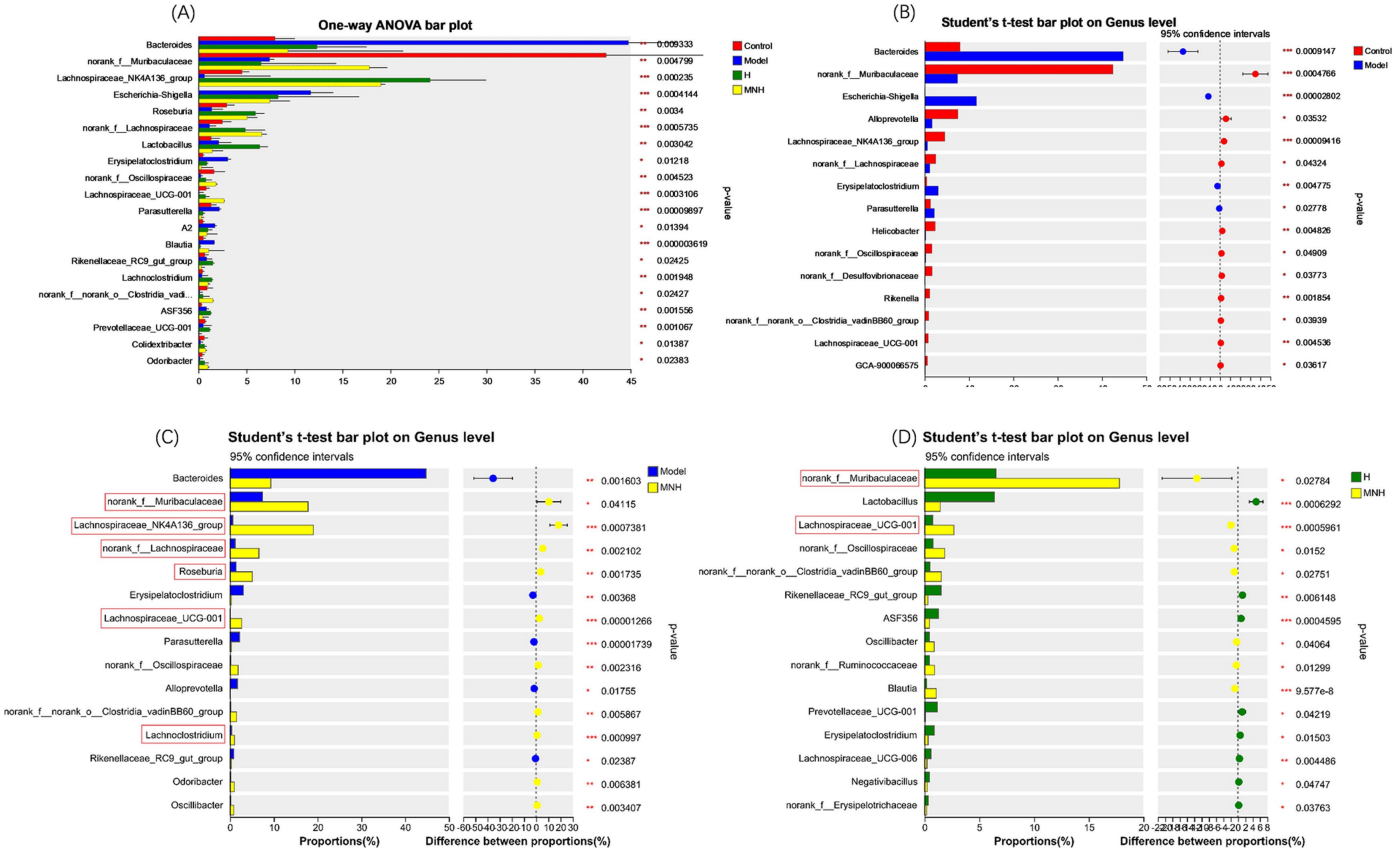
Comparison of fecal gut microbiota at genus level between groups. **(A)** Model, H, MNH, and control groups. **(B)** Control vs. model. **(C)** MNH vs. model. **(D)** MNH vs. H groups. ^*^p < 0.05, ^**^p < 0.01, and ^***^p < 0.001.

To further analyze the differences in the flora of each group, a one-way ANOVA was performed on the abundance of the flora (Figure 6A). The results showed that Bacteroides, Escherichia-Shigella, Erysipelatoclostridium, and Parasutterella were increased in abundance in mice with AA compared to the control group; Lachnospiraceae_NK4A136_group, norank_f__Lachnospiraceae, and Lachnospiraceae_UCG-001 were reduced in abundance in mice with AA, and it was considered that an increase in these opportunistic pathogens and a decrease in the beneficial bacteria might contribute to AA pathogenesis (Figure 6B). These beneficial bacteria increased after microneedling treatment while pathogenic bacteria showed a significant downward trend. However, the improvement of elevated Escherichia-Shigella in the model group was not statistically significant, so microneedling treatment may be able to improve the gut microbe disorders due to AA. In addition, microneedling treatment increased the norank_f_ Muribaculaceae, Lachnospiraceae_NK4A136_group, norank__f__Lachnospiraceae, Roseburia, Lachnospiraceae_UCG-001, and Lachnoclostridium groups (Figure 6C) and increased the abundance of norank_f__Muribaculaceae and Lachnospiraceae_UCG-001 compared to treatment with halometasone alone (Figure 6D).

### 3.6 Effect of microneedling therapy on fecal SCFAs in AA mice

**Figure 7:**
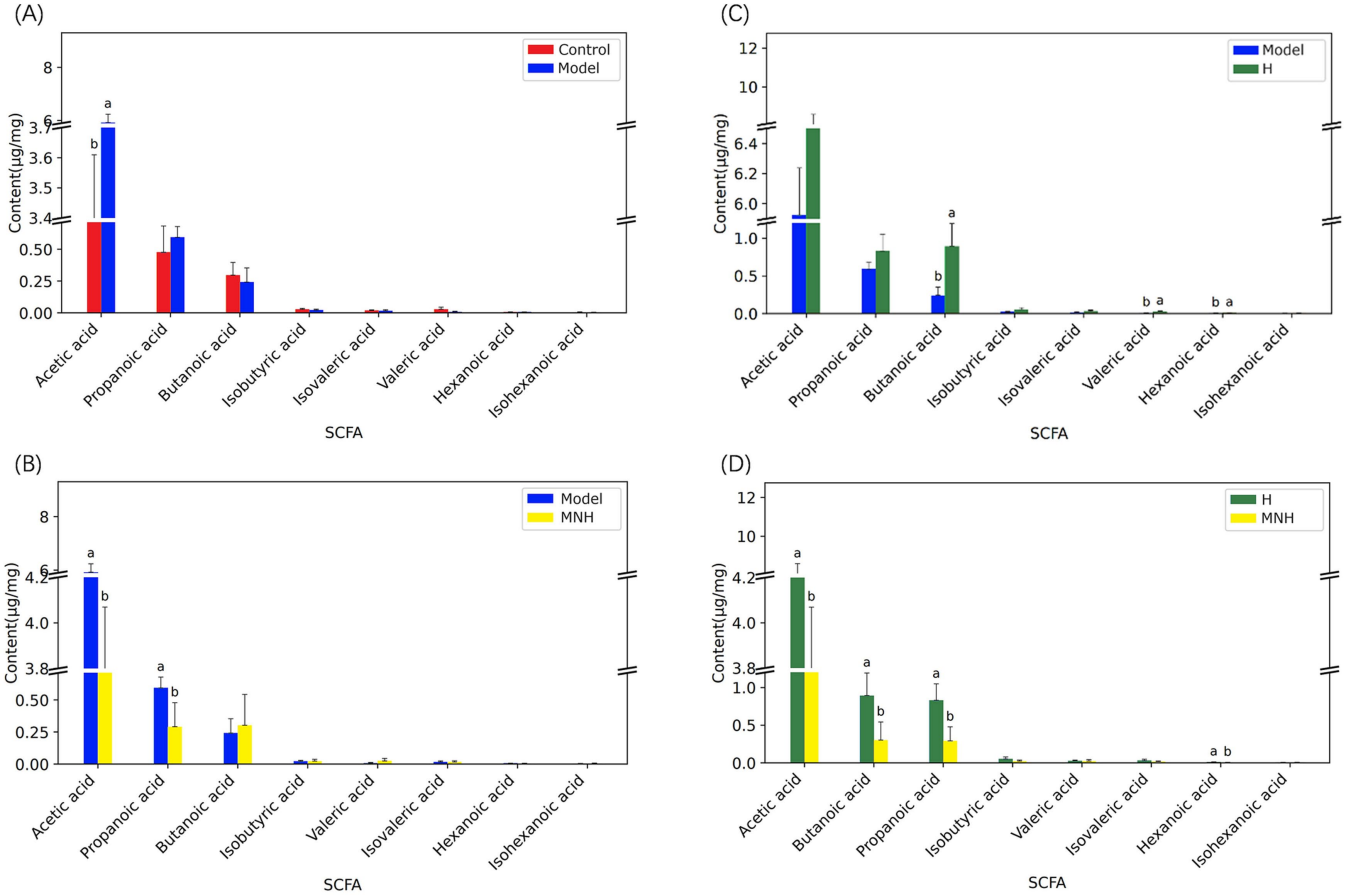
Comparison of short-chain fatty acids among groups. **(A)** Control group and model group. **(B)** Model group and H group. **(C)** Model group and MNH group. **(D)** H group and MNH group. For the same indicator, if the letters of the two groups are the same, there is no significant difference in the content of the indicator in the two groups.

Acetic acid and propanoic acid were elevated in the feces of mice in the model group, and the levels were significantly down-regulated after microneedling treatment. At the same time, microneedling treatment also upregulated butyric acid in the feces of mice in the model group, but the difference was not statistically significant. Microneedling treatment had no significant effect on the levels of isobutyric acid, isovaleric acid, isohexanoic acid, valeric acid, or hexanoic acid (Figures 7A–D).

## 4 Discussion

Gut microbes, also called the ‘second genome,’ represent a dynamic ecosystem within the human body (Grice and Segre, 2012). Dysbiosis of the gut microbes can result in disorders of intestinal microbial metabolism, which, in turn, can lead to immune dysfunction (Ni et al., 2022). The study of the gut-skin axis has highlighted the role of gut flora on skin health. These microbial communities are important in maintaining the gut-skin dynamic balance (O’Neill et al., 2016; Ellis et al., 2019). Disruption in the relationship between the gut flora and the immune system may impact the skin, potentially leading to the development of skin diseases. There is a robust correlation between gut flora dysbiosis and the development of inflammatory dermatological conditions, including psoriasis, acne, seborrheic dermatitis, atopic dermatitis, and AA (Gao et al., 2023). Our findings revealed that the development of AA and microneedling treatment in mice resulted in alterations to the composition of the gut microbes.

It is currently believed that AA is an autoimmune disease associated with Th1 (IL-2, IFN-γ, TNF, IL-12, and IL-18), Th2 (IL-4, IL-5, IL-6, IL-9, IL-10, IL-13, IL-17E, IL-31, and IL-33), Th17 (IL-17, dysregulation of IL-17F, IL-21, IL-22, IL-23, and TGF-β), and Treg (IL-35) type cytokines. These cytokines are associated with the pathogenesis, clinical manifestations, and prognosis of AA (Waśkiel-Burnat et al., 2021; Yang et al., 2024). Our findings revealed that AA changed the gut microbes of mice, with a notable reduction in diversity and abundance compared to the control group. The abundance of Bacteroides, Escherichia-Shigella, Erysipelatoclostridium, and Parasutterella was increased in mice with AA. Holmes et al. (2023) found that patients with AA have an increased risk of atopic and autoimmune comorbidities, which supports previous studies that suggest that Th1- and Th2-type inflammatory responses may be implicated in the pathogenesis of AA. Bacteroides is associated with autoimmune disorders (Jin et al., 2023). The Bacteroidaceae and its sub-taxon Bacteroides have been identified as risk factors for atopic dermatitis. The abundance of Bacteroides is significantly higher in patients with atopic dermatitis than in healthy individuals, with the proportion positively correlating with disease severity (Mahdavinia et al., 2019; Han et al., 2022). Lipopolysaccharide (LPS), a metabolite of Bacteroides, can induce the production of inflammatory cytokines by acting on Toll-like receptor 4 (TLR4) on the surface of cell membranes. There is a high degree of synergy between LPS and IL-4-polarized macrophages, which promote a Th2-type inflammatory response in the organism (Curtis et al., 2014; Czimmerer et al., 2022). Erysipelatoclostridium is a bacterium that induces inflammation and leads to dysregulation of the intestinal microecological balance (Zhang N. et al., 2023). In a study by Nagayama et al. (2020), the colonization of germ-free mice with nine strains, including Erysipelatoclostridium, was observed to promote the aggregation of Th1-type cells and, to a lesser extent, the accumulation of Th17-type cells in the intestine. Parasutterella is an opportunistic pathogen that disrupts the homeostasis of organismal immune cells (Shin et al., 2015). Studies have demonstrated that Parasutterella can accumulate in mice, resulting in the loss of immune cells in the small intestine in a non-inflammatory manner (Zheng et al., 2021). There is a strong correlation between AA and inflammatory bowel disease (IBD), which exhibit shared immune and genetic pathways (Maghfour et al., 2021). Similarly, interferon-gamma (IFN-γ) and tumor necrosis factor-alpha (TNF-α) play a pivotal role in the pathogenesis of AA. There is a positive correlation between Parasutterella and the exacerbation of IBD (Chen et al., 2018). Mice with inflammatory bowel disease exhibit elevated levels of pro-inflammatory cytokines (IL-1, IL-6, and TNF-α) and decreased levels of anti-inflammatory cytokines (IL-13) in serum. These changes are accompanied by an increased abundance of gut microbes, specifically Bacteroides and Parasutterella (Zheng et al., 2021). This illustrates that alterations in the gut microbes may influence the levels of inflammation-related cytokines in the body, thereby contributing to the onset of inflammatory bowel disease. Our study also indicated that these genera were predominantly elevated in mice with AA. Nevertheless, it is important to recognize that differences still exist in other specific genera when compared to inflammatory bowel disease. Disturbances in the gut microbes or changes in specific microbiota ultimately result in the development of autoimmune diseases by participating in the body’s inflammatory response, secreting various inflammatory cytokines, and modulating the host’s immune status. It can thus be proposed that gut microbes dysbiosis may be involved in the pathogenesis of AA in mice.

The results of our study demonstrated that microneedling treatment also resulted in alterations to the species composition of the gut microorganisms in AA mice, with a notable increase in the abundance of Bacteroidaceae. Yang et al. (2023) postulated that four bacterial species, including the Muribaculaceae, maybe the key flora for promoting hair follicle growth. Their findings revealed a significant increase in abundance in otter rabbits with very high follicle densities. Furthermore, the skin of these otters exhibited markedly elevated expression of WNT4, WNT10a, WNT10b, CTNNB1 (β-catenin), and LEF1. These findings align with those of our study. Microneedling has been demonstrated to stimulate the expression of Wnt protein, which promotes hair follicle development and hair growth through the Wnt/β-catenin signaling pathway (English et al., 2022; Shin, 2022). Furthermore, the F/B value of gut microbes was observed to decrease in AA mice and subsequently increase following microneedling treatment. An elevated or decreased F/B ratio has been demonstrated to induce an imbalance in the intestinal microecological environment. The former will result in obesity and metabolic disorders, which may be attributed to an increase in calorie intake, fat metabolism, and impaired insulin sensitivity. Conversely, the latter will lead to inflammatory bowel disease, depression, and Alzheimer’s disease, which may be associated with SCFAs, particularly butanoic acid. A reduction in production may also contribute to an immune-inflammatory response due to the accumulation of protein metabolites, including histamine and LPS (Stojanov et al., 2020; Mariat et al., 2009; Grigor’eva, 2020; Vaiserman et al., 2020).

The metabolism of flora in the intestine can produce SCFAs, which mainly contain acetic acid, propanoic acid, butanoic acid, isobutyric acid, valeric acid, isovaleric acid, hexanoic acid, and isocaproic acid. These play important roles in maintaining the integrity of the intestinal mucosa, balancing the metabolic functions of the body, and regulating the immune response (Zhang D. et al., 2023). The production of acetic, propanoic, and butanoic acid by gut microbial metabolism accounts for over 90% of SCFAs (Kimura et al., 2020).

Butanoic acid represents the primary source of energy for intestinal cells, playing an essential role in maintaining intestinal homeostasis and safeguarding the functional integrity of the intestinal barrier. It can stimulate intestinal cell proliferation, promote nutrient absorption, and inhibit the body’s inflammatory response and oxidative stress (Sitkin and Pokrotnieks, 2019). Concurrently, butanoic acid can regulate the immune system and peripheral immune tolerance through the up-regulation of regulatory T cells (Tregs) and the down-regulation of macrophages (Jacob et al., 2020). A reduction in butanoic acid-producing Clostridium has been observed in patients with AA compared to healthy controls (Moreno-Arrones et al., 2020; Lu et al., 2021).

The present study demonstrated that both halometasone and microneedling therapy increased the abundance of Roseburia and four other genera within the Lachnospiraceae family. Notably, microneedling therapy resulted in a more pronounced increase in the abundance of Lachnospiraceae within the gut microbes. Furthermore, the microneedling treatment also upregulated reduced butanoic acid in the metabolites of the gut flora of AA mice. Lachnospiraceae is a potentially beneficial bacterium involved in the metabolism of a wide range of carbohydrates and is an important butanoic acid-producing bacterium. Pyrophosphate sequencing experiments have demonstrated that butanoic acid-producing bacteria are of particular importance for the maturation of the host immune system (Jost et al., 2013).

Roseburia is a member of the Lachnospiraceae family and represents an important genus of beneficial bacteria within the gut microbiota. As a genus that produces a substantial amount of butanoic acid, Roseburia plays a pivotal role in regulating inflammatory processes. Butanoic acid has been demonstrated to reduce LPS-induced TNF-α and nitric oxide synthase (Nos) expression in monocytes, thereby exerting anti-inflammatory effects through the activation of G-protein-coupled receptors involved in apoptosis and natural immunity-related processes (Calzadilla et al., 2023; Li et al., 2018). Some researchers have hypothesized that butanoic acid may safeguard hair follicles from immune assault by inducing immune tolerance in regulatory T cells (Treg) through the stimulation of GPR43, GPR41, or GPR109 (Fertig et al., 2018). Roseburia typically demonstrates net acetate consumption and elevated butyrate production during carbohydrate metabolism (Tamanai-Shacoori et al., 2017). The results of the present study demonstrated that acetic acid was elevated in the feces of AA mice, with significantly reduced levels observed following microneedling treatment. This may be related to the metabolism of Roseburia.

Propanoic acid has been shown to protect the organism by destroying bacteria and viruses and play a crucial role in lipid metabolism, the nervous system, and cardiovascular diseases (Zhang D. et al., 2023). However, the results of the present study demonstrated that microneedling therapy significantly downregulated the elevated levels of propanoic acid in the metabolites of the gut microbes of AA mice. Ormsby et al. (2020) observed in a Crohn’s disease-associated mouse model that high concentrations of propanoic acid led to the development of resistance and increased virulence in pathogenic *E. coli*. The results of our study demonstrated an increase in the abundance of Escherichia-Shigella in AA mice, which was subsequently observed to decrease after microneedling treatment. As one of the most prevalent intestinal pathogens, Shigella can directly invade epithelial cells, damaging the intestinal barrier and disrupting the host’s immune homeostasis. Furthermore, Shigella can metabolize LPS, disrupting the intestinal microecological balance (Vega-Magaña et al., 2018). While propanoic acid has been demonstrated to possess immunomodulatory properties within the gut, this is a particularly pertinent consideration in the context of disease. Although propanoic acid can reduce disease incidence by signaling to specific immune cells, propanoic acid-driven phenotypic switching can also result in bacterial overgrowth (Smith et al., 2013).

In addition, we found that the level of beneficial bacterial genera such as Lachnospiraceae_NK4A136_group, norank__f__ Lachnospiraceae, and Lachnospiraceae_UCG-001 was decreased during the process of AA formation in mice, and the level of beneficial bacterial genera was significantly improved after microneedling treatment. It suggested that the change in the level of beneficial flora caused by AA was corrected after microneedling treatment, and the change in the gut microbes after microneedling treatment may be favorable to the growth of hair in AA.

In summary, the results of 16S rRNA analysis showed that AA can lead to a decrease in the diversity and abundance of gut microbes, with significant changes in the structure of some flora and changes in the content of SCFAs. Microneedling therapy can improve the reduced abundance of gut microbes caused by AA than conventional therapy, resulting in a significant increase in the relative abundance of beneficial flora, change in intestinal metabolites SCFAs, and the promotion of intestinal mucosal barrier recovery. However, further in-depth studies on the role and mechanism of SCFAs in the AA intestinal tract are needed. This provides new ideas for the pathogenesis and treatment options of AA. Microneedling therapy may be an alternative to conventional therapies for AA, while targeted therapies for gut microbes and SCFAs may also be beneficial for patients with AA.

## Data Availability

The original contributions presented in the study are publicly available. This data can be found here: https://www.ncbi.nlm.nih.gov, accession number PRJNA1303621.
